# Effectiveness of the Histone Deacetylase Inhibitor (S)-2 against LNCaP and PC3 Human Prostate Cancer Cells

**DOI:** 10.1371/journal.pone.0058267

**Published:** 2013-03-04

**Authors:** Anna Laurenzana, Manjola Balliu, Cristina Cellai, Maria Novella Romanelli, Francesco Paoletti

**Affiliations:** 1 Department of Experimental Pathology and Oncology, University of Florence, Firenze, Italy; 2 Department of Pharmaceutical Sciences, University of Florence, Sesto Fiorentino, Italy; Albany Medical College, United States of America

## Abstract

Histone deacetylase inhibitors (HDACi) represent a promising class of epigenetic agents with anticancer properties. Here, we report that (S)-2, a novel hydroxamate-based HDACi, shown previously to be effective against acute myeloid leukemia cells, was also a potent inducer of apoptosis/differentiation in human prostate LNCaP and PC3 cancer cells. In LNCaP cells (S)-2 was capable of triggering H3/H4 histone acetylation, H2AX phosphorylation as a marker of DNA damage and producing G_0_/G_1_ cell cycle arrest. Consistently, (S)-2 led to enhanced expression of both the protein and mRNA p21 levels in LNCaP cells but, contrary to SAHA, not in normal non-tumorigenic prostate PNT1A cells. Mechanistic studies demonstrated that (S)-2-induced apoptosis in LNCaP cells developed through the cleavage of pro-caspase 9 and 3 and of poly(ADP-ribose)-polymerase accompanied by the dose-dependent loss of mitochondrial membrane potential. Indeed, the addition of the pan-caspase inhibitor Z-VAD-fmk greatly reduced drug-mediated apoptosis while the antioxidant *N*-acetyl-cysteine was virtually ineffective. Importantly, preliminary data with nude mice xenografted with LNCaP cells showed that (S)-2 prompted a decrease in the tumor volume and an increase in H2AX phosphorylation within the cancer cells. Moreover, the highly metastatic prostate cancer PC3 cells were also sensitive to (S)-2 that: i) induced growth arrest and moderate apoptosis; ii) steered cells towards differentiation and neutral lipid accumulation; iii) reduced cell invasiveness potential by decreasing the amount of MMP-9 activity and up-regulating TIMP-1 expression; and iv) inhibited cell motility and migration through the Matrigel. Overall, (S)-2 has proven to be a powerful HDACi capable of inducing growth arrest, cell death and/or differentiation of LNCaP and PC3 prostate cancer cells and, due to its low toxicity and efficacy *in vivo*, might also be of clinical interest to support conventional prostate cancer therapy.

## Introduction

Epigenetic changes are reversible chromatin rearrangements capable of modulating gene expression within the cell without modifying DNA sequence. Acetylation is the most widely studied post-translational modification of histone proteins [1] due to the balanced activity of two families of enzymes, namely the histone acetyltransferases (HATs) and histone deacetylases (HDACs) that catalyze the acetylation/deacetylation of histones, respectively, and thereby modifying chromatin conformation and DNA accessibility to transcription factors [2], [3], [4]. Moreover, HATs and HDACs contribute to modulating gene expression by direct interaction with nonhistone key regulatory proteins [5] as p53, GATA1, GATA2, retinoic acid receptor, NF-kB and cytoskeletal proteins like α-tubulin [6], [7], [8]. It is not surprising, therefore, that aberrant activities of these enzymes may repress transcription of specific onco-suppressor genes and lead, eventually, to tumor formation [9], [10]. And indeed, histone hypoacetylation, due to over-expression of HDACs, has a recognized role in the tumorigenesis of different cancers affecting stomach [11], colon [12], [13], breast [14] and prostate [15], [16], [17].

With particular regard to prostate cancer, this is the most frequently diagnosed non-cutaneous malignancy and the third leading cause of cancer-related deaths in men in the Western world. Although a number of therapeutic options are available for early prostate cancer, in patients relapsed from primary treatment with surgery and/or radiation, or presenting metastatic disease, the androgen deprivation remains the mainstay of therapy. However, despite the androgen ablation, virtually all tumors eventually progress with castration-resistant diseases [18], [19], [20] which need to be treated with conventional cytotoxics or epigenetic agents such as HDAC inhibitors (HDACi). The latter have emerged as a new class of powerful anticancer agents capable of inducing tumor cell growth arrest, differentiation and/or apoptosis [16], [17] *in vitro* and acting as radiation sensitizers in cancer cells by down-regulating DNA repair activity [21], [22], [23]. Some of these HDACi showed however several limitations *in vivo* due to their high toxicity, low solubility, and short half-lives [24], [25]. Therefore, developing novel HDACi with anticancer properties and low-toxic profiles is a crucial target of translational research.

We have previously reported a new set of potent hydroxamate-based HDACi characterized by a 1,4-benzodiazepine ring (BDZ) used as the cap and linked, through a triple bond connection unit, to a linear alkyl chain carrying a hydroxamic function as the Zn^++^-chelating group [26]. Among these hybrids, one in particular, MC133(S)-2 [henceforth (S)*-*2] showed to be a very effective pro-apoptotic agent towards different cultured and primary acute myeloid leukemia (AML) cells *in vitro* and *ex vivo*, and was virtually safe to mice *in vivo* up to 150 mg/kg/week [27].

In the present study we examined the antitumor potential of (S)-2 in two of the most widely investigated human epithelial prostate cancer cell lines, namely the androgen-sensitive LNCaP, and the androgen-insensitive and highly metastatic PC3, by using the human nontumorigenic PNT1A prostate epithelial cells as the control. (S)-2 inhibited prostate cancer cell proliferation, induced a greater apoptotic response as compared to SAHA (or Vorinostat; one of the best performing HDACi approved by FDA for treatment of cutaneous T-cell lymphoma) [28], [29] in LNCaP cells and to a lesser extent also in highly metastatic PC3 cells whose migration and invasiveness properties were drastically reduced by the drug. In contrast, normal epithelial prostate PNT1A cells were virtually drug insensitive. Importantly, (S)-2-induced apoptosis in LNCaP cells developed through a caspase-dependent mechanism.

## Materials and Methods

### Cell Culture and Treatments

Nonmetastatic LNCaP and metastatic PC3 prostate cancer cells, and the human nontumorigenic prostate epithelial PNT1A cells were a kind gift of P. Chiarugi (Dept. Biochemical Sciences, University of Florence) who obtained the cell lines from the European Collection of Cell Cultures [30]. Human prostate cells were cultured in RPMI 1640 supplemented with 10% fetal bovine serum (FBS). Cells were maintained at 37C° in 5% CO_2_ humidified atmosphere. (S)-2 and SAHA (or Vorinostat; Sigma-Aldrich, St. Louis, MO, USA) [28], [29] were dissolved in dimethyl sulfoxide (DMSO; Sigma-Aldrich) at 0.1 M concentration and stored in the dark at room temperature (RT). Working drug solutions were obtained by appropriate dilution of the stock solution with the culture medium. DMSO was employed as the vehicle at a final dose of ≤0.1% (v/v) in culture for both (S)-2 and SAHA. In caspase inhibition experiments Z-VAD-fmk (R&D Systems, Minneapolis, MN, USA) and the anti-oxidant N-Acetyl Cysteine (NAC; Sigma-Aldrich) were added in culture 2 h prior to (S)-2 addition.

### Cell Cycle Analysis

Prostate cells were treated for 24 h without/with 2.5 µM drug, then resuspended in a propidium iodide/RNase solution (BD PharMingen, San Diego, CA) and incubated at RT in the dark for 15–30 min. The percentages of cells relative to G_0_/G_1_, G_2_/M, and S phase were determined with the aid of Becton Dickinson FACSCalibur System.

### Western blotting

Harvested cells were resuspended in 20 mM RIPA buffer (pH 7.4) containing a cocktail of proteinase inhibitors (Calbiochem, Merck, Darmstadt, Germany) and treated by sonication (Microson XL-2000, Minisonix, Farmingdale, NY, USA). Proteins were assayed by the BCA Protein Assay (Thermo Scientific, Rockford, IL, USA), analysed by SDS-PAGE and western blotting as reported elsewhere [31]. Membranes were probed with primary antibodies against: acetyl-H3, acetyl-H4, and H4 (Upstate Biotechnology, Millipore, Bilerica, MA, USA); PARP, γ-H2AX, H2AX, H3 and Caspase 9 (Cell Signaling Technology, Danvers, MA, USA); α-tubulin and acetylated α-tubulin (Sigma-Aldrich), Caspase 3 and p21 (Santa Cruz Biotechnology, Santa Cruz, CA, USA). Suitable peroxidase-conjugated IgG preparations (Sigma-Aldrich) have been used as secondary antibodies; the ECL procedure was employed for development.

### Quantitation of Mitochondrial Membrane Potential

To determine changes in drug-induced transmembrane mitochondrial membrane potential (Δψm), cells have been stained with JC-1 (Invitrogen, Life Technologies, Carlsbad, CA, USA), a cationic dye that exhibits potential-dependent accumulation in mitochondria, indicated by a fluorescence emission shift from green (525±10 nm) to red (610±10 nm). LNCaP cells (0.5×10^6^) were treated without/with 2.5 and 5 µM (S)-2 for 72 h and then resuspended in RPMI 1640 containing 15 µg/ml of JC-1 dye for 30 min at RT in the dark; after that cells were washed and the fluorescence was measured by flow cytometry. Mitochondria depolarization is specifically indicated by a decrease in the red to green fluorescence intensity ratio [32].

### Caspase 3 Activation Assay

Prostate cancer cells (10^5^ cell/ml) were incubated with 2.5 µM (S)-2 for 48 h and then subjected to the Carboxyfluorescein FLICA Apoptosis Detection Kit Caspase assay (Caspase 3 FLICA, Immunochemistry Technology, Bloomington, MN, USA). Cells were stained with FAM-DEVD-FMK FLICA reagent dissolved in PBS for 1 h at 37°C, and washed twice in PBS before performing the cytofluorimetric assay.

### Gel Zymography

Analysis of gelatinase (MMP-9) activity was performed as previously described [33]. Briefly, PC3 cells were seeded in 6-well plates and treated with increasing amount of (S)-2 in serum-free media for 24 h. Aliquots of conditioned media (CM) were mixed with 4× (v/v) sample buffer (0.25 mol/l Tris-HCl, pH 6.8, 0.4% SDS, 40% glycerol and bromophenol blue), then loaded onto a 10% SDS gel containing 1 mg/ml gelatin (Sigma-Aldrich) and run under non-reducing conditions at the constant voltage of 125 V. Following electrophoresis, the gel was incubated in renaturing buffer (2.5% Triton X-100) at RT for 30 min, washed twice with distilled water (10 min each time), and then incubated with the developing buffer (50 mmol/l Tris pH 8.0, 5 mmol/l CaCl_2_, 0.2 mol/l NaCl and 0.02% Brij-35) at 37C° overnight, stained in 0.5% Coomassie Blue solution for 2 h and destained with a solution [5% acetic acid, 10% methanol (v/v) in distilled water] until bands of gelatinolytic activity were visualized and then measured by densitometric analysis with Image J Software.

### Wound-healing Assay

PC3 cells were cultured in 6-cm plates until confluence and then the monolayer was scratched using a fine sterile pipette tip to produce a narrow wound in the substrate. The medium and debris were aspirated away and replaced with fresh medium in the presence of different concentrations of (S)-2. Pictures were taken before and 24 h after wounding with the aid of a Nikon E 4500 photocamera (Nikon) on a Nikon TMS-F phase-contrast microscope (Nikon Instruments, Florence, Italy).

### Invasiveness Assay

For these experiments were used Boyden chambers in which the upper and lower wells were separated by porus polycarbonate filters coated with matrigel (50 µg/filter) (Becton Dickinson, BD, New Jersey, USA). Cultures were pre-treated with/without (S)-2 (2.5–5 µM) for 24 h and then aliquots of PC3 cells (20×10^3^) were transferred in the upper compartment of the chamber. Cell invasive capability was evaluated after 6 h and expressed as the absolute number ± SD of cells present on the filters; five different microscopic fields for each condition have been examined.

### Oil Red O Staining for Neutral Lipids

Neutral lipids were detected (i) histochemically [34] on cell monolayers which were quickly fixed with?−?0°C methanol, stained with Oil Red O (ORO) (Sigma-Aldrich), and (ii) spectrophotometrically (Cary 50 Scan, Varian, Victoria, Australia) at 510 nm by recording absorbance of cell-bound ORO following extraction with isopropanol [35], [36]. ORO accumulation was expressed as relative absorbance unit/mg cell protein.

### Quantitative Real Time-PCR Analysis

QRT-PCR was performed with reverse transcripted cDNA of untreated and treated cells using the Applied Biosystems 7500HT System according to standard protocols. Fold of p21, MMP-9 and TIMP-1 induction were calculated by the changes of p21 or MMP-9 or TIMP-1 Ct values in treated *versus* untreated cells and were normalized to the 18S rRNA Ct Amplification was performed with the default PCR setting: 40 cycles of 95°C for 15 sec and of 60°C for 60 sec using a SYBR Green based detection (SYBR Green Master mix; Applied Biosystems) and the following primers: for p21, forward 5′-CTGCCCAAGCTCTACCTTCC-3′?and reverse 5′-CAGGTCCA CATGGTCTTCCT-3′; for MMP-9 forward 5′-GCTACCACCTCGAA CTTTGAC-3′ and reverse 5′-TGCCGGATGCCATTCAC-3′; for TIMP-1, forward 5′-CCAACAG TGTAGGTCTTGGTGAAG-3′ and reverse 5′-CTGTGGCTCCCTGGAACA-3′; and for 18S rRNA, forward 5′CGGCTACCACATCCAAGGAA-3′?and reverse 5′-GCTGGAATTACCGCGGCT-3′.

### Preliminary *in vivo* Experiments with a Murine Xenograft Model

The protocol and results regarding this preliminary approach to *in vivo* experiment has been reported under the section of Supporting Information (Figure S1).

### Statistical Analysis

The Student’s *t*-test or one-way analysis of variance were used to assess statistical significance of results. The difference among the values was considered significant at *P* ≤ 0.05.

## Results

### (S)-2 Prompts LNCaP Cells to G_0_/G_1_ Cell Cycle Arrest and Changes in Morphology

**Figure 1:**
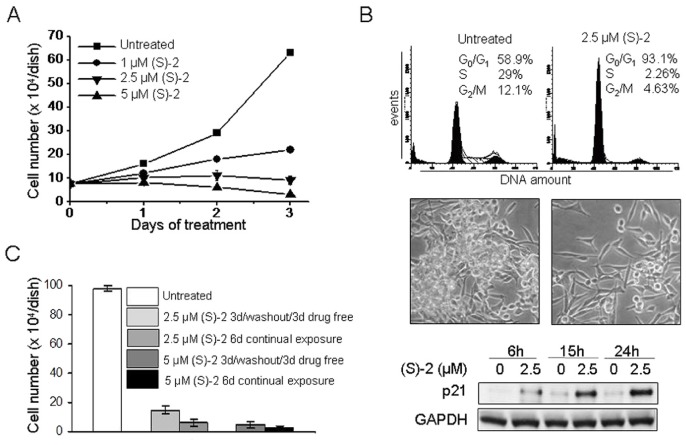
(S)-2 induced growth arrest in LNCAP cells. (**A**) – Cells (10^5^) were seeded in 6-well plates and allowed to attach overnight. On the next day (S)-2 was added at the indicated concentrations (0–5 µM) and viable cells (trypan blu-negative) were counted with the aid of a Bürker chamber along the following three days. (**B**, top) – (S)-2 induced G_0_/G_1_ cell cycle arrest and increased p21 expression. LNCaP cells (2×10^5^) were treated with 2.5 µM drug for 24 h, then were detached and aliquots of cell suspensions were incubated with a propidium iodide (PI) solution for 30 min and subsequently analyzed by flow cytometry (DNA amount, X-axis; total events, Y-axis). The percentage of cells in the different phases of the cell cycle was calculated by the ModFit program and shown in each panel. (**B**, middle) – Phase contrast pictures of companion cultures indicated that (S)-2 induced morphological changes and a marked decrease in cell density. (**B**, bottom) – Cells were treated with 2.5 µM drug for the indicated time points and p21 protein levels were monitored by immunoblotting; GAPDH was also examined to ensure equal loading of samples in each lane. (**C**) – LNCaP cell growth arrest was not strictly dependent on the continual presence of drug. Cells have been seeded in 6-well plates (10^5^ cell/well) and allowed to attach overnight. The day after cultures were added without/with 2.5–5 µM (S)-2 for 3d and then replaced with drug-free medium for additional 3d and compared to cultures where the drug was steadily maintained up to 6d when viable cells were counted. Each bar represents the mean obtained from triplicate wells ± SD.

LNCaP cells, cultured without/with increasing (S)-2 concentrations (1, 2.5 and 5 µM) up to 72 h, underwent a dose- and time-dependent growth arrest to reach 50% of inhibition after two days of incubation with 1–2.5 µM (S)-2; whereas in cultures treated with 5 µM the number of viable cells decreased significantly to levels well below the starting plating density (Figure 1A). The effect of (S)-2 on LNCaP cell cycle progression as measured by flow cytometry showed that a 24 h-exposure to 2.5 µM drug increased significantly the percentage of cells in G_0_/G_1_ (from 59 to 93%) and decreased cell population in S-phase (from 29 to 2%) (Figure 1B, top). In addittion, upon treatment, the typical morphology of LNCaP cells changed to a spindle-shaped, fairly enlarged phenotype to yield monolayers that were characterized by a partial cell loss and reduced contacts among residual cells (Figure 1B, middle). Consistently, the cell cycle inhibitor p21 protein – reported to be up-modulated by HDACi [37] – augmented in a time-dependent manner in response to 2.5 µM (S)-2; the protein was detected starting from 6 h of treatment, increased after 15 h and peaked at 24 h (Figure 1B, bottom).

Furthermore, growth arrest of LNCaP exposed to (S)-2 was not strictly dependent on the continual presence of drug. This assumption derived from monitoring cell number in cultures treated without/with 2.5 or 5 µM (S)-2 for 3d and then replaced with drug-free medium for additional 3d, while in companion cultures the drug was steadily maintained for 6d. Cells remained virtually arrested even after the drug-free medium replacement yielding values which were similar to those of cultures continually exposed to the drug (Figure 1C).

### (S)-2-induced Apoptosis in LNCaP Cells Depends on Activation of the Caspase Cascade and Disruption of Mitochondrial Integrity

**Figure 2:**
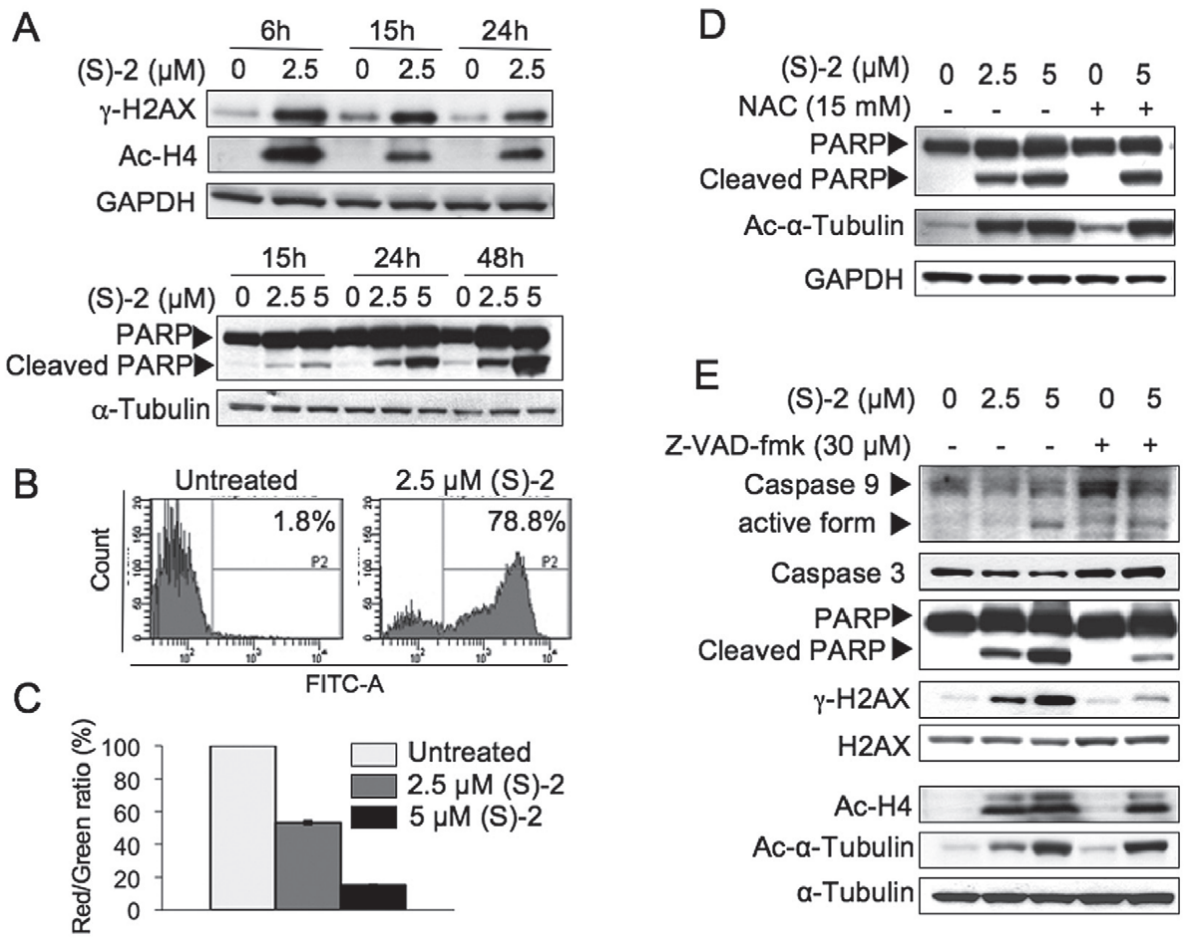
(S)-2 induced apoptosis in LNCaP cells. (**A**) **–** Cells were incubated with the drug (2.5, 5 µM) for the indicated time points. Whole-cell extracts were analysed by Western immunoblot to detect: phospho-H2AX, that it is triggered following DNA damage; PARP and its cleaved fragment to denote apoptotic activaction; and acetyl-H4 due to the inhibition of HDAC activity. GAPDH and α-tubulin were used as loading controls. (**B**) **–** Untreated or drug-treated cells (2.5 µM for 48 h) were incubated during the the last 60 min with FAM-DEVD-FMK carboxyfluoresceine, then rinsed twice with PBS and their green fluorescence was measured by flow cytometry. The frequency histogram of the number of events (Y axis) *versus* fluorescein intensity (X axis) showed two peaks: caspase-negative cells (unlabeled cells) were on the left of the P2 region; while caspase-positive cells which were labeled with Flica occurred within the P2 region. (**C**) – Treatment with (S)-2 led to a dose-dependent mitochondrial transmembrane potential (ΔΨ) dissipation. This effect was assessed with the aid of JC-1 dye, which aggregates in normal mitochondria and emits red fluorescence but it can not accumulate in mitochondria which have lost their transmembrane potential, and, therefore emits a diffuse cytoplasmatic green signal in dead cells. Mitochondrial depolarization is indicated by a decrease in the red/green (R/G) fluorescence intensity ratio [32]. Values have been normalized by using the control signal (only the vehicle) as an arbitrary value of 100%. Each bar is the mean of two independent experiments performed in triplicate. (**D**) **–** LNCAP cells were treated with (S)-2 (2.5–5 µM) or with (S)-2 (5 µM) plus 15 mM N-Acetyl Cysteine (NAC) applied 2 h before drug addition. Activation of apoptosis was revealed by the cleavage of PARP and phosphorylation of H2AX and these events as well as the drug-mediated α-tubulin acetylation were not contrasted by NAC. GAPDH was used as the reference protein. (**E**) – Z-VAD-fmk prevented the drug-induced cleavage of PARP and the phosphorylation of H2AX. LNCAP cells were treated as above but, instead of NAC, cultures were preincubated with 30 µM Z-VAD-fmk for 2 h prior to be treated for 24 h with the drug. Cell lysates were analyzed for the cleavage of PARP, the activation of caspase 3 and 9, H2AX phosphorylation as well as acetylation of H4 and α-tubulin; α-tubulin was used as loading control.

Among the early HDACi-induced events at DNA level there were the formation of double strand breaks (DSB) and the phosphorylation of H2AX to yield γ-H2AX that aids in DNA damage repair [38]. The response of LNCaP cells to (S)-2 as determined by immunostaining showed that 2.5 µM drug significantly increased the γ-H2AX signal within 6 h and these levels were fairly sustained up to 24 h by following a pattern similar to that of drug-induced acetyl-H4 (Figure 2A, top). Moreover, the onset of apoptosis, as marked by cleavage of the caspase substrate poly(ADP-ribose) polymerase (PARP), was detected from 15 h of treatment and increased steadily up to 48 h (Figure 2A, bottom) when about 80% of LNCAP cells showed the: (i) drug-mediated activation of caspase 3 (Figure 2B) and (ii) dose-dependent shift in the JC-1 red/green fluorescence ratio to denote a progressive dissipation of the mitochondrial transmembrane potential (ΔΨm) (Figure 2C).

Moreover, to investigate the mechanism of (S)-2-induced apoptosis in LNCAP cells, the effects of the anti-oxidant N-acetyl-cysteine (NAC) and of the pan-caspase inhibitor Z-VAD-fmk were examined separately. Differently from that reported for AML cells [27], the presence of 15 mM NAC in the culture medium was not capable of decreasing drug-induced cleavage of PARP thus ruling out a major role of reactive oxygen species (ROS) in drug-mediated apoptosis (Figure 2D). Instead, experiments performed without/with 30 µM pan-caspase inhibitor Z-VAD-fmk revealed that this compound was capable of preventing drug-mediated activation of caspase 9 and 3 as well as of the cleavage of PARP and increase in γ-H2AX [39], thus suggesting that (S)-2-induced apoptosis in LNCaP cells developed through a caspase-dependent mechanism (Figure 2E). It is worth noting that drug-induced acetylation of H4 and α-tubulin was not hampered by Z-VAD-fmk.

### (S)-2 Targets LNCaP Cells but not Normal Prostate PNT1A Cells

**Figure 3:**
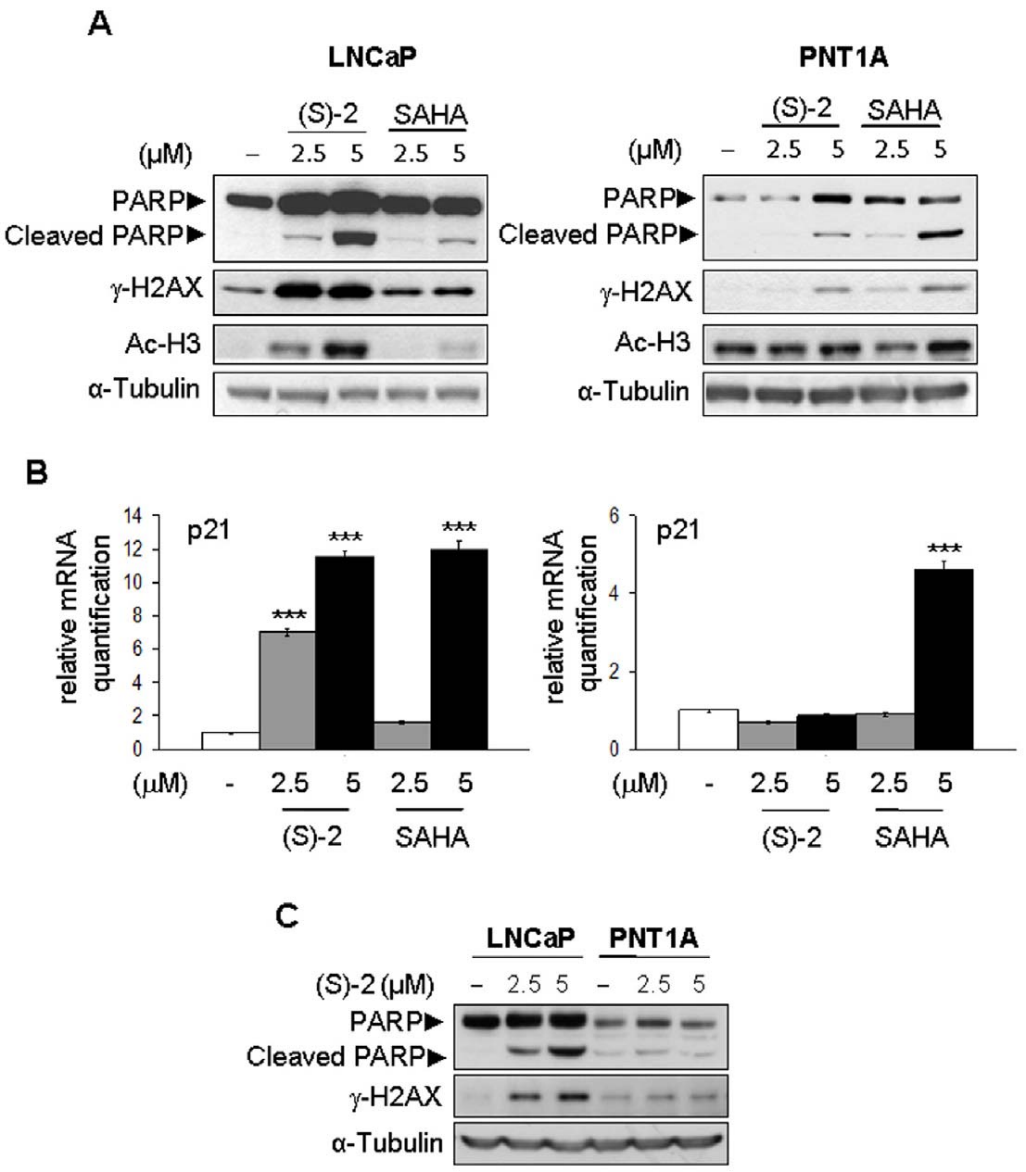
Effects of (S)-2 and SAHA towards LNCAP and PNT1A cells. (**A**) – LNCAP and PNT1A cells were incubated for 24 h with increasing amounts of either (S)-2 and SAHA. Cell extracts were subjected to Western immunoblotting to detect phospho-H2AX, PARP and its cleaved fragment and acetyl-H3; α-tubulin was used as loading control. (**B**) **–** p21 mRNA levels from LNCAP and PNT1A cells incubated without/with (S)-2 or SAHA for 24 h were measured by quantitative real-time PCR. Normal PNT1A cells were apparently less sensitive to (S)-2 as compared to SAHA. Columns, average of three independent samples: bars ± SD; significant difference (*P*≤0.05). (**C**) **–** PARP cleavage and γ-H2AX levels induced in LNCaP and PNT1A cells by a 24 h-treatment without/with (S)-2 were compared on the same blot.

The potential translational value of (S)-2 was assessed by comparing the activities of both (S)-2 and SAHA with regard to induction of apoptosis and histone acetylation on LNCaP cells and normal prostate epithelial immortalized PNT1A cells. (S)-2 prompted a marked increase in the levels of cleaved PARP fragment, γ-H2AX and acetyl-H3 in LNCaP cells with much greater efficacy than SAHA (Figure 3A, left). Moreover, (S)-2 seemed to be relatively safe to normal PNT1A cells that, instead, were a sensitive target of SAHA as revealed by the cleavage of PARP upon treatment with 5 µM drug (Figure 3A, right) while acetyl-H3 levels in PNT1A cells remained relatively steady regardless of either the inducers.

Furthermore, growth arrest in (S)-2-treated LNCAP cells was associated with a marked dose-dependent increase (7–13 times) in p21 mRNA levels which were also enhanced by SAHA although with a less dose-dependent progression (Figure 3B, left). It should be noted that p21 expression in PNT1A cells was unaffected by (S)-2, while strikingly up-regulated by 5 µM SAHA (Figure 3B, right). Moreover, results obtained by comparing on the same blot the effects of (S)-2 in LNCaP and PNT1A cells have clearly proven that LNCaP were definitely more sensitivity than normal PNT1A cells in terms of PARP cleavage and γ-H2AX levels (Figure 3C).

### (S)-2 Induces Cell Cycle Arrest, Apoptosis and Differentiation of PC3 Cells

**Figure 4:**
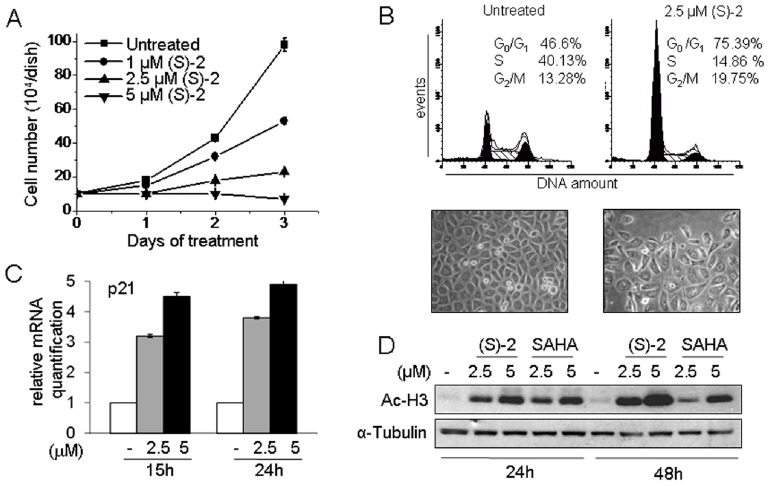
PC3 cells undergo growth arrest upon treatment with (S)-2. (**A**) **–** Cells (10^5^) were seeded in 6-well plates and allowed to attach overnight. The day after (S)-2 was added at the indicated concentrations and cell number was determined along the next three days. (**B**) – Cells were incubated for 24 h with 2.5 µM (S)-2 to determine the % of PI-stained cells in different phases of the cell cycle as determined by flow cytometry. Pictures of either untreated and treated cultures were taken with the aid of a phase-contrast microscopy. (**C**) **–** p21 mRNA levels in PC3 cells from cultures treated without/with (S)-2 were measured by quantitative real-time PCR. (**D**) **–** Comparative Western blot analysis of acetyl-H3 levels in cell extracts from PC3 treated with either (S)-2 or SAHA; α-tubulin was used as loading control.

The effect of (S)-2 on proliferation of the highly metastatic prostate cancer PC3 cells has also been evaluated. Cells cultured for three days without/with increasing amounts of (S)-2 underwent a dose-dependent inhibition of proliferation (Figure 4A) in keeping with the significant increase in the proportion of cells arrested in G_0_/G_1_ phase (from 46 to 75%) and the decrease (from 40 to 15%) of cells in S phase (Figure 4B). Consistently, p21 was significantly up-regulated at 15 and 24 h of treatment (Figure 4C). Of interest, (S)-2-induced acetyl-H3 levels were already enhanced at 24 h and rose further at 48 h, just when the effect of SAHA began to decline (Figure 4D).

**Figure 5:**
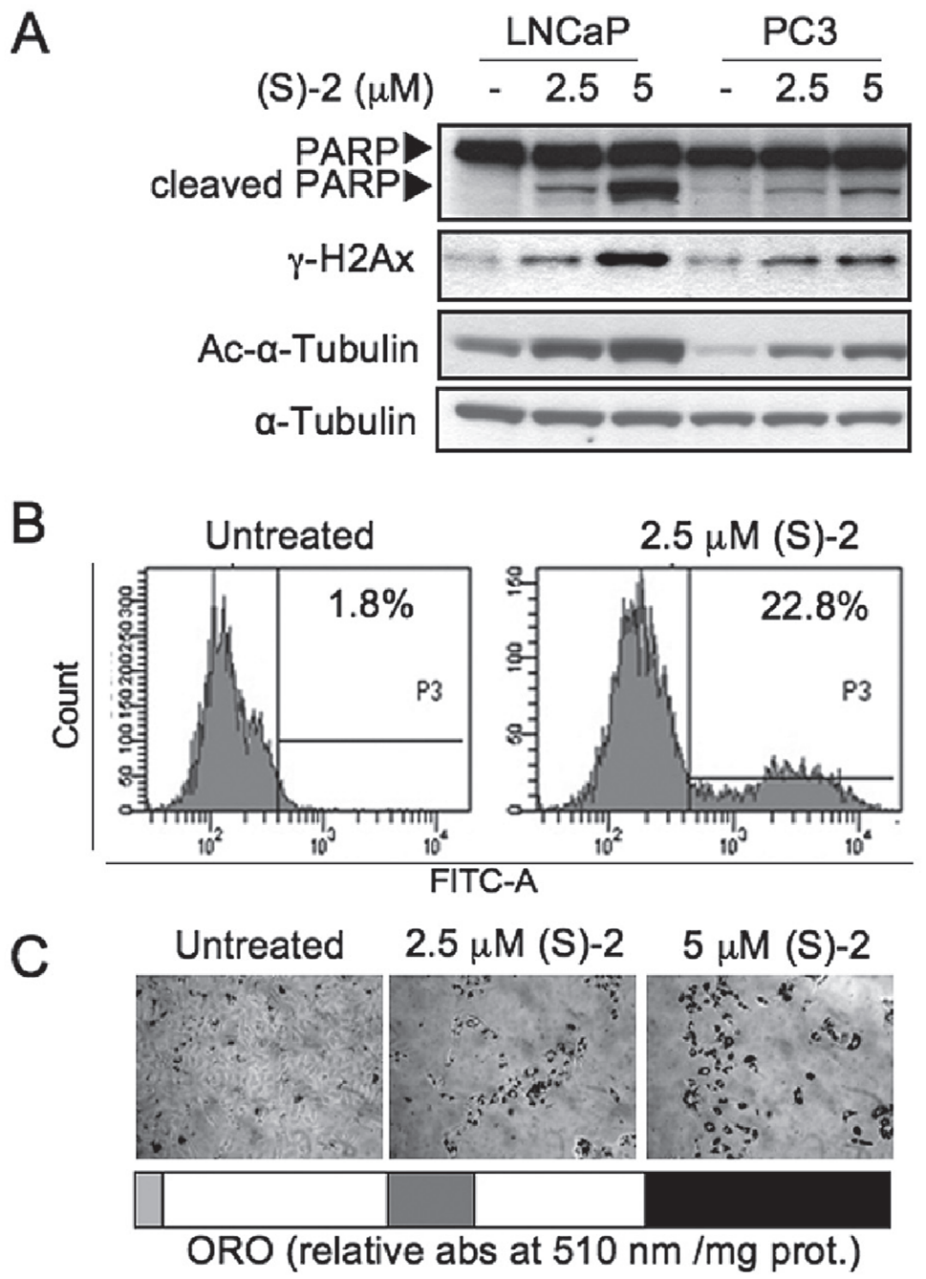
PC3 are less sensitive than LNCAP cells to drug-induced apoptosis. (**A**) – Samples from PC3 and LNCaP cells treated without/with (S)-2 (2.5 and 5 µM) for 24 h were analysed by Western blot and immunodetected for: PARP and its cleaved fragment, γ-H2AX and acetyl-α-tubulin, while α-tubulin was used as loading control. (**B**) – Cells either untreated or treated with 2.5 µM (S)-2 for 48 h were incubated just 1 h prior to be harvested with FAM-DEVD-FMK carboxyfluoresceine and then rinsed twice with PBS and their green fluorescence was measured by flow cytometry (see comment of point B in Figure 2). (**C**) – Microscopic evaluation of the effects of (S)-2 on the accumulation of neutral lipid droplets within PC3 cells treated for three days. After fixation, cells were stained with an ORO solution; quantification of ORO staining was carried out as described in Materials and Methods.

However, PC3 cells, despite their sensitivity to (S)-2-mediated cytostasis, seemed to be more resistant than LNCaP cells to drug-induced apoptosis as shown by the fact that a similar pattern of cleaved PARP, γ-H2AX and acetyl-α-tubulin in the two cell lines could be obtained only by treating PC3 cells with twice the dosage employed for LNCaP cells (Figure 5A). Furthermore, the fluorescent assay for caspase 3 activation by 2.5 µM (S)-2 indicated that about 23% of PC3 cells underwent apoptosis after a 48 h-treatment (Figure 5B) *i.e.* less than one-third relative to treated LNCAP cells. In addition, PC3 cells remaining on the dish following incubation for 72 h with increasing amounts of drug became larger in size relative to controls and accumulated within the cytoplasm neutral lipid droplets, staining positively with Oil-Red O (ORO) as the result of drug-induced adipogenic differentiation (Figure 5C) that was already reported to occur in these cells [40].

### (S)-2 Reduces Invasiveness, Migration and Motility of PC3 Cells

**Figure 6:**
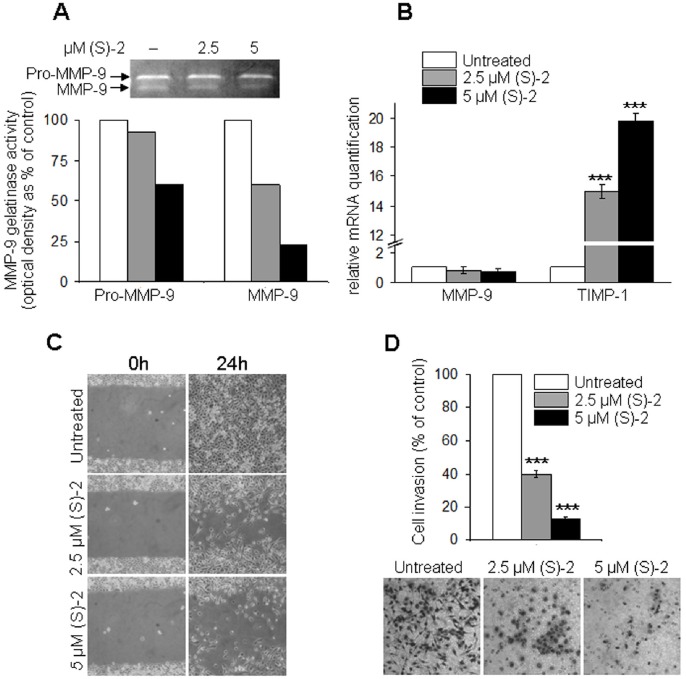
(S)-2 reduces invasiveness, migration and motility potential of PC3 cells. (**A**) **–** Aliquots of conditioned media from PC3 cultures incubated without/with (S)-2 in the absence of FCS were submitted to gelatin zymography and densitometric analysis of MMP-9 activity (percentage of control). (**B**) **–** MMP-9 and TIMP-1 mRNA levels from PC3 cells treated without/with (S)-2 for 24 h were determined by quantitative real-time PCR. (**C**) **–** (S)-2 inhibited PC3 cell motility *in vitro*. Confluent cultures were “wounded” with the aid of a sterile plastic tip and maintained without/with increasing amounts of drug for 24 h. A phase-contrast microscopy was used to take pictures of the monolayers. (**D**) – (S)-2 decreased invasiveness of PC3. Cultures were pre-treated with/without (S)-2 (2.5–5 µM) for 24 h and then aliquots of PC3 cells (20×10^3^) were transferred in the upper compartment of the chamber. Cells migrated through the Matrigel on the filters of Boyden chambers were counted after 6 h and expressed as the absolute cell number ± SD; five different microscopic fields (magnification: x200) for each condition were examined and significant difference among specimens was established at *P*≤0.05.

Matrix metalloproteinases (MMPs) released by tumor cells into the extracellular environment are crucial for cancer-promoted tissue degradation and invasion along with the metastatic process [41], [42], [43]. MMP-9 from the conditioned medium of PC3 cultures was submitted to gelatin zymography and showed a dose-dependent decrease of MMP-9 activity (Figure 6A) that was accompanied by a slight, yet not significant, decline in MMP-9 expression (Figure 6B, left). Instead, the expression of the tissue inhibitor of metalloproteinase-1 (TIMP-1) – known to exert anti-metastatic effects by contrasting the activity of MMP-9 and other MMPs [44], [45] – was strikingly enhanced after 24 h of treatment (Figure 6B, right).

Moreover, results of the “wound healing” assay *in vitro* showed that in untreated cultures the wounded area was completely refilled within 24 h, while in drug-treated cultures cell migration and motility was decreased in a dose-dependent manner (Figure 6C). Finally, the invasive potential of PC3 cells through the Matrigel was markedly inhibited after 24 h of incubation with increasing (S)-2 concentrations as shown by the number of stained cells on the filters of Boyden chambers and related pictures (Figure 6D).

## Discussion

Anticancer properties of the novel HDACi (S)-2 on LNCaP and PC3 prostate carcinoma cells have been evaluated in this work by using the PNT1A cells as the normal prostate epithelial counterpart and SAHA as the prototype of hydroxamate-based HDACis. (S)-2 exerted a broad spectrum of effects towards prostate cancer cells including G_0_/G_1_ cell cycle arrest, p21 up-regulation, histone hyperacetylation and induction of apoptosis as showed by the caspase cascade activation, the cleavage of PARP, the disruption of mitochondrial integrity and DNA damage. These events, as thouroughly explained in the previuos section, were prompted by low micromolar drug dosages and within a relatively short time (6–72 h). Thus, rather than reiterate specific experimental details, it is useful to address a few, but important points.

First, (S)-2 proves to be a more effective agent than SAHA as concerns the extent and duration of drug-induced PARP cleavage activity in both LNCaP and PC3 cells as well as its safety towards normal epithelial prostate PNT1A cells. Moreover, SAHA was reported to recognize the androgen-dependent prostate LNCaP and CWR22 cells as sensitive targets, but not the androgen-independent PC-3 and TSU-Pr1 cells which showed a modest drug-induced growth arrest with a little detectable cell death [46].

Second, mechanistic studies indicated that (S)-2-induced apoptosis in LNCaP cells was mainly based on caspase cascade activation. Conversely, pro-apoptotic effects of (S)-2 in different AML cell types, developed through a ROS-dependent mechanism [27]. This might suggest that (S)-2 is able to effectively trigger apoptosis in both hematological (AML) and solid prostate carcinoma (LNCaP) cells although *via* two distinct pathways. On the other hand, SAHA-induced apoptosis in LNCaP, but not in PC3 cells [46], was ascribed to drug-induced ROS accumulation and, consistently, was significantly contrasted by thioredoxin [47].

Third, nonmetastatic LNCaP cells were apparently more sensitive than metastatic PC3 cells to (S)-2-induced apoptosis. However, this does not mean that the drug was poorly effective on PC3 cells, but, more properly, that PC3 response to (S)-2 is the combined result of multiple different processes that, in addition to partial growth arrest and apoptosis, also include: (i) changes in cell morphology and neutral lipid accumulation in residual cells as a part of drug-induced differentiation [40], and (ii) most importantly, a striking drug-induced inhibition in cell invasion, motility and migration through the Matrigel, which are crucial steps in tumor progression and dissemination of metastatic cells, and consistent with the increase in acetylated α-tubulin that it is present in stable microtubules but is absent from dynamic cellular structures [48]. Overall, these events are, prospectively, more significant for contrasting highly metastatic and drug-resistant cells like p53-null PC3 rather than for nonmetastatic cells such as LNCaP for which a clnical treatment might be easier and with a more favourable outcome.Furthermore, both (S)-2 and SAHA share the same hydroxamic function as a zinc-chelating group but differ in the cap portion: SAHA has a small achiral hydrophilic anilido group whereas in (S)-2 there is a bulky lipophilic 5-phenyl-1,4-benzodiazepin-3-one ring carrying a stereogenic center important for activity [26], [27]. Also, in (S)-2, linker and cap are joined through a metabolically stable triple bond that is typical of oxamflatin [49], while in SAHA these two domains are connected through an hydrolysable amide bond. Looking at these chemical differences, it is not surprising that (S)-2 and SAHA display distinct biological efficacy and action mechanisms in prostate and other cancer cells.

And finally, preliminary experiments with nude mice xenografted with LNCaP cells showed that (S)-2 was capable of reducing the tumor volume and increasing γ-H2AX levels within tumor cells *in vivo* as the best evidence for drug-induced caspase activation and DNA damage (Figure S1).

On the whole, (S)-2 showed to be a safe HDACi *in vivo* [27] with powerful anti-proliferative, pro-apototic and differentiative properties towards nonmetastatic and higly metatastatic prostate carcinoma cells, making this agent of potential clinical interest in support of conventional therapy for this and, possibly, other types of cancer.

## Supporting Information

Figure S1 **Preliminary** ***in vivo*** **experiments with a murine xenograft model.** Male nude (nu/nu) athymic mice (Harlan Laboratories, Srl, San Pietro al Natisone, UD, Italy) were cared for and maintained in accordance with applicable European Animal Welfare regulations under an approved Institutional Animal Care and Use Protocol in an animal facility at University of Florence accredited by the Association for Assessment and Accreditation of Laboratory Animal Care. Aliquots of LNCaP cell suspension (3×10^6^ cell/100 µl containing an equal volume of RPMI and matrigel) were implanted subcutaneously on the left flank of 12 mice [50]. A week later the tumor mass was present only in eight out of the originally injected mice (66% of tumor incidence) which were then randomized into two equal groups and drug treatment was started. (S)-2 was formulated as a DMSO solution and injected intraperitoneally. One group was treated with the drug (50 µl final volume, at 65 mg/kg, corresponding to approximately 2 mg/mouse) three times a week for two weeks, while the control group was treated with 50 µl DMSO alone as the vehicle. Mice were killed after 6 treatments and 24 h post-dose by cervical dislocation. (**A**) – Tumors were measured by calipers after the sacrifice (bottom); the tumor mass was weighed and the volume was calculated according to the formula length × width^2^ × π/6 (top). In all cases, tumor volumes in untreated mice were significantly larger relative to those of drug-treated mice to suggest that (S)-2 was capable of reaching the cancer cells and decreasing their growth rates. Photographs are representative of a tumor mass from a mouse treated with either the vehicle or (S)-2, respectively. (**B**) – For immunohistochemistry, slides with 2.5–5 µm sections of paraffin embedded tumor mass were first deparaffinized, boiled in 1 mM EDTA pH 9 for 15 min and after cooling aspecific peroxidases were blocked with 3% H_2_O_2_ for 10 min. Then, slides were according to standard procedures and incubated with a primary antibody against γ-H2AX (see Materials and Methods) followed by a peroxidase-conjugated IgG preparation; 3,3′-diaminobenzidine was used as the chromogen for development. Slides were counterstained with aqueous Meyer hematoxylin and mounted with glycerol for visual inspection and photography; pictures are representative of four randomly chosen microscopic fields (magnification: x400). γ-H2AX-positive cells were frequently observed within the tumor mass of drug-treated mice but not of mice injected with the vehicle only as depicted by the pictures (bottom) and clearly indicated by the histograms (top). Statistical analysis was carried out by Student’s *t*-test and significant differences between the two groups were indicated by the asterisks (*P<0.05; ***P<0.001). Importantly, as reported elsewhere in CD-1 mice [27], no specific drug-induced histologic alteration in May-Grünwald Giemsa-stained liver parenchymal cells was observed (data not shown).
(EPS)
